# Metabolite-mediated responses of phyllosphere microbiota to powdery mildew infection in resistant and susceptible black currant cultivars

**DOI:** 10.1093/hr/uhaf092

**Published:** 2025-03-25

**Authors:** Xueying Zhao, Along Chen, Xiaonan Gong, Peng Zhang, Kaojia Cui, Shuxian Li, Weixia Zhang, Chenqiao Zhu, Huixin Gang, Junwei Huo, Fuchun Xie, Dong Qin

**Affiliations:** College of Horticulture & Landscape Architecture, Northeast Agricultural University, No 600, Changjiang Road, Xiangfang District, Harbin City, Heilongjiang Province, China; National-Local Joint Engineering Research Center for Development and Utilization of Small Fruits in Cold Regions, Northeast Agricultural University, No 600, Changjiang Road, Xiangfang District, Harbin City, Heilongjiang Province, China; College of Horticulture & Landscape Architecture, Northeast Agricultural University, No 600, Changjiang Road, Xiangfang District, Harbin City, Heilongjiang Province, China; Shandong Provincial Forestry Protection and Development Service Center, No.5948, Second Ring East Road, LixiaDistrict, Jinan City, Shandong Province, China; College of Horticulture & Landscape Architecture, Northeast Agricultural University, No 600, Changjiang Road, Xiangfang District, Harbin City, Heilongjiang Province, China; National-Local Joint Engineering Research Center for Development and Utilization of Small Fruits in Cold Regions, Northeast Agricultural University, No 600, Changjiang Road, Xiangfang District, Harbin City, Heilongjiang Province, China; College of Horticulture & Landscape Architecture, Northeast Agricultural University, No 600, Changjiang Road, Xiangfang District, Harbin City, Heilongjiang Province, China; National-Local Joint Engineering Research Center for Development and Utilization of Small Fruits in Cold Regions, Northeast Agricultural University, No 600, Changjiang Road, Xiangfang District, Harbin City, Heilongjiang Province, China; College of Horticulture & Landscape Architecture, Northeast Agricultural University, No 600, Changjiang Road, Xiangfang District, Harbin City, Heilongjiang Province, China; National-Local Joint Engineering Research Center for Development and Utilization of Small Fruits in Cold Regions, Northeast Agricultural University, No 600, Changjiang Road, Xiangfang District, Harbin City, Heilongjiang Province, China; College of Horticulture & Landscape Architecture, Northeast Agricultural University, No 600, Changjiang Road, Xiangfang District, Harbin City, Heilongjiang Province, China; National-Local Joint Engineering Research Center for Development and Utilization of Small Fruits in Cold Regions, Northeast Agricultural University, No 600, Changjiang Road, Xiangfang District, Harbin City, Heilongjiang Province, China; College of Horticulture & Landscape Architecture, Northeast Agricultural University, No 600, Changjiang Road, Xiangfang District, Harbin City, Heilongjiang Province, China; National-Local Joint Engineering Research Center for Development and Utilization of Small Fruits in Cold Regions, Northeast Agricultural University, No 600, Changjiang Road, Xiangfang District, Harbin City, Heilongjiang Province, China; College of Horticulture & Landscape Architecture, Northeast Agricultural University, No 600, Changjiang Road, Xiangfang District, Harbin City, Heilongjiang Province, China; National-Local Joint Engineering Research Center for Development and Utilization of Small Fruits in Cold Regions, Northeast Agricultural University, No 600, Changjiang Road, Xiangfang District, Harbin City, Heilongjiang Province, China; College of Horticulture & Landscape Architecture, Northeast Agricultural University, No 600, Changjiang Road, Xiangfang District, Harbin City, Heilongjiang Province, China; National-Local Joint Engineering Research Center for Development and Utilization of Small Fruits in Cold Regions, Northeast Agricultural University, No 600, Changjiang Road, Xiangfang District, Harbin City, Heilongjiang Province, China; College of Animal Science and Technology, Northeast Agricultural University, No 600, Changjiang Road, Xiangfang District, Harbin City, Heilongjiang Province, China; College of Horticulture & Landscape Architecture, Northeast Agricultural University, No 600, Changjiang Road, Xiangfang District, Harbin City, Heilongjiang Province, China; National-Local Joint Engineering Research Center for Development and Utilization of Small Fruits in Cold Regions, Northeast Agricultural University, No 600, Changjiang Road, Xiangfang District, Harbin City, Heilongjiang Province, China

## Abstract

Plant–metabolite–microbe interactions play essential roles in disease suppression. Most studies focus on the root exudates and rhizosphere microbiota to fight soil-borne pathogens, but it is poorly understood whether the changes in phyllosphere metabolites can actively recruit beneficial microbes to enhance disease resistance. In this study, the differences of phyllosphere microbial communities and key leaf metabolites were systematically explored in resistant and susceptible black currant cultivars related to powdery mildew (PM) by integrating microbiome and metabolomic analyses. The results showed that the diversity and composition of microbiome changed, as highlighted by a reduction in microbial alpha-diversity and beta-diversity of susceptible cultivars. An increasing fungal network complexity and a decreasing bacterial network complexity occurred in resistant cultivar. *Bacillus*, *Burkholderia* (bacteria), and *Penicillium* (fungi) were identified as keystone microorganisms and resistance effectors in resistant cultivar. Metabolites such as salicylic acid, *trans*-zeatin, and griseofulvin were more abundant in resistant cultivar, which had a positive regulatory effect on the abundance of bacterial and fungal keystones. These findings unravel that resistant cultivar can enrich beneficial microorganisms by adjusting leaf metabolites, thus showing the external disease-resistant response. Moreover, the reduced stomatal number and increased tissue thickness were observed in resistant cultivar, suggesting inherent physical structure also provides a basic defense against PM pathogens. Therefore, resistant black currant cultivar displayed multilevel defense responses of physical structures, metabolites, and microorganisms to PM pathogens. Collectively, our study highlights the potential for utilizing phyllosphere microbiome dynamics and metabolomic adjustments in agricultural practices, plant breeding, and microbiome engineering to develop disease-resistant crops.

## Introduction

Black currant (*Ribes nigrum* L.) is a perennial deciduous shrub in the Saxifragaceae family, renowned for its high ascorbic acid content (350 mg/100 g fresh weight) [1, 2]. This berry possesses significant nutritional and medicinal value, particularly as a unique species in the alpine regions of Northeast China [3]. One of the most serious threats to black currant production is powdery mildew (PM), a prevalent fungal disease caused by obligate parasitic fungi [4]. These pathogens infiltrate the host plant to absorb nutrients, significantly affecting plant health. Research has identified two primary PM pathogens in *Ribes*: *Podosphaera mor-uvae* Berk. *et* Curt and *Sphaerotheca macularis*, both belonging to the Policaceae family [5]. Powdery mildew primarily targets the leaves, stems, and young fruits of black currant. In severe cases, the infection can spread throughout the entire plant. Infected leaves exhibit a characteristic white mold layer, which eventually turns gray-brown as the lesions expand. Due to the PM pathogen invading through the leaf undersides, controlling the disease presents tremendous challenges. While chemical pesticides can inhibit PM development, they entail high economic costs and pose a threat to human health and the environment. Thus, there is a pressing need to explore sustainable and environmentally friendly strategies to enhance the plant’s adaptive abilities against PM infection.

The plant-associated microbiota is referred to as the second genome of the plant [6]. Mutualistic interactions between plants and microbiota have huge impacts on plant nutrient acquisition [7], disease resistance [8], and complex environment adaptation [9]. A growing body of evidence shows that plants enhance their resistance against pathogens by recruiting beneficial microbes from the environment or reshaping the microbial community composition [10, 11]. For example, chili pepper (*Capsicum annuum*) infected with *Fusarium* wilt disease could attract beneficial bacteria and alleviate the changes in reproductive organ microbiome to support the viability of its offspring [12]. Additionally, multicycle wheat plantings and pathogen infection could significantly influence the composition of rhizosphere microbial communities, leading to an enrichment of beneficial microorganisms that suppress pathogens and foster crop growth [13]. Most studies focused on the effective strategies of rhizosphere microbiota in response to various diseases [14, 15], but there remains a limited understanding of the resistance mechanism of the microbial community in the phyllosphere against pathogen infection. In addition, plant species and genotypes are important determinants of the microbial community structure, as well as certain species and genotypes that generally form stronger associations with specific microbial taxa [16, 17]. In particular, plant cultivars with different resistance to certain pathogens can harbor distinct microbial community compositions. Resistant and susceptible tomato cultivars (*Solanum lycopersicum*) demonstrate differences in their capacity to recruit beneficial microbes upon *Fusarium oxysporum* f. sp. *lycopersici* (FOL) infection [18]. Likewise, the disease-resistant common bean cultivars promote the enrichment of specific bacterial species in the rhizosphere to suppress pathogens [19], which indicates that particular microbes could play protective roles in helping plants cope with pathogen infections. The distinct abilities of resistant and susceptible cultivars in recruiting beneficial microbes to suppress pathogen infection have crucial implications, but the mechanistic explanation remains largely unclear.

Plant metabolites have been identified as biochemical barriers to resist pathogen attacks and can participate in the transduction of disease resistance response as signal substances [20]. Studies revealed that specific metabolites can impact the composition and function of microbiota to facilitate plant defense [21–23]. In two *Malus* species, certain metabolites have been found to promote the recruitment of phyllosphere microbiota in response to rust infection [24]. Benzoxazinoids affect specific microbial clusters to enhance defense responses in maize [25]. Similarly, secondary metabolite fusaric acid induced tomato rhizosphere microbiota recruitment and altered Fusarium wilt disease resistance [18]. Several organic compounds and other molecules such as flavonoids, coumarins, and phytohormones have been regarded as plant signals that are involved in the microbial community [26–28]. It has been reported that polyamine [29], resveratrol [30], callose [31], salicylic acid (SA) [32], and jasmonic acid (JA) [33] were important metabolites involved in responses to PM. However, not much is known about the extent and mechanism of metabolites regulating the plant microbiomes and whether such changes could contribute to plant responses against PM infection.

It was found that the varying resistance levels of different black currant cultivars to PM were quite different in production, which may be associated with the microbial population structure and metabolites on the leaf surface. However, it is still unclear how the microbiome and metabolites related to the aerial surface of black currant enhance PM resistance. Therefore, in the current study, PM-resistant ‘16A’ and susceptible ‘Bright leaf’ were used to analyze metabolite differences and the structure of phyllosphere microbial communities via high-throughput 16S rRNA, internal transcribed spacer (ITS) sequencing, and metabolome. We aimed to explore the regulatory effect of leaf metabolites on recruiting beneficial microbes and identify candidate microbes that are significantly associated with PM resistance. This was the first report revealing the mechanisms of phyllosphere metabolites and microbes related to PM resistance in *Ribes*. The findings would be helpful to reveal the mechanisms of plant–metabolite–microbe interactions for resisting PM, and provide valuable insights for the evaluation and breeding of PM resistance germplasm resources.

## Results

### Morphological observation and molecular identification of powdery mildew

The black currant leaves were the main tissue infected by PM, and the infected leaves had whitish colonies on the surfaces. Optical microscopic observations revealed that conidiophore was vertically attached to the hypha, with an unbranched size of 53.7~69.4 × 8.6~5.4 μm, and conidia were strung on it (Fig. S1A). Conidia produced after maturity were oval or nearly spherical, with a size of 21.8–30.3 × 11.3–18.5 μm (Fig. S1B). Conidia were colorless and had many fibrous bodies inside. When conidia germinated, the bud tube protruded from one side (Fig. S1C). Wintering in the form of closed capsule shell (Fig. S1D). The morphological characteristics of the PM pathogen in Heilongjiang province was typical of the *P. mor-urae*.

**Figure 1:**
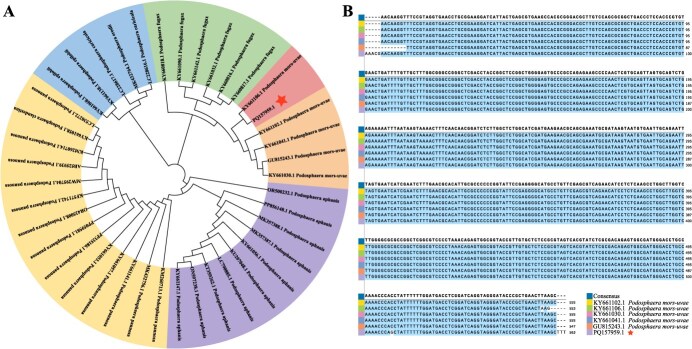
**Molecular analysis and identification of PM pathogen in black currant.** (A) Phylogenetic tree based on the ITS sequences derived from different plant species. The ITS sequence in this study was indicated by red stars. (B) Sequences comparison of PM pathogen.

The ITS sequence of the pathogenic fungus was amplified by polymerase chain reaction (PCR) to obtain a 563-bp sequence (GenBank accession number: PQ157959.1). Phylogenetic tree was constructed based on the ITS sequences. A high level of sequence similarity (98%) was observed between the pathogen isolated in this study and that on *Podosphaera mors-uvae* in *Ribes sanguineum* (Fig. 1A). The ITS sequences from Heilongjiang province were compared with those identified previously as *P. mors-uvae* of other species (Fig. 1B), and the results further proved that the pathogenic fungus of black currant PM in Heilongjiang province was *P. mor-uvae*.

### Leaf structural differences between resistant and susceptible black currant

**Figure 2:**
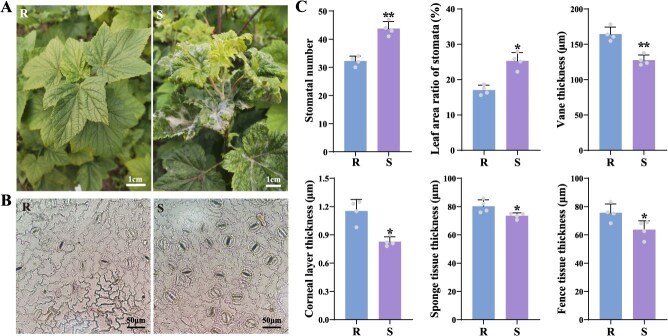
**Differences in phenotype and leaf structure of the resistant and susceptible black currant cultivars**. (A) Morphological characteristics. (B) Lower epidermal stomata. (C) Comparison of leaf structures. The bars represented standard deviation. Statistical significance was indicated by asterisks (^*^P < 0.05, ^**^P < 0.01). R: resistant cultivar ‘16A’, S: susceptible cultivar ‘Bright leaf’.

To investigate the effects of leaf structure on PM pathogen tolerance, PM-resistant ‘16A’ (R) and susceptible ‘Bright leaf’ (S) of black currant were used for the study (Fig. 2A). Stomata is an important channel for pathogens to infect plants, and the number, size, and structure of stomata affect the success rate of pathogen invasion. Average stomatal number of S was significantly higher than that in the R (*P* < 0.01, Fig. 2B and C). The stomatal size of PM-resistant and susceptible cultivars was similar. However, due to the different stomatal density, the leaf area ratio of stomata in resistant cultivar was less than that of susceptible cultivar. There was prominent difference in leaf thickness between PM-resistant and susceptible black currant (*P* < 0.01, Fig. 2C). Resistant cultivar showed significantly higher cuticle thickness, sponge tissue thickness, and fence tissue thickness compared to susceptible cultivar (*P* < 0.05, Fig. 2C). Microscopy observation showed that the palisade tissue cells of resistant cultivar were arranged more tightly and neatly (Fig. S2). This suggested that plants with thick leaf tissue were more resistant to pathogen infection.

### Differential metabolomic responses of black currant cultivars to powdery mildew

**Figure 3:**
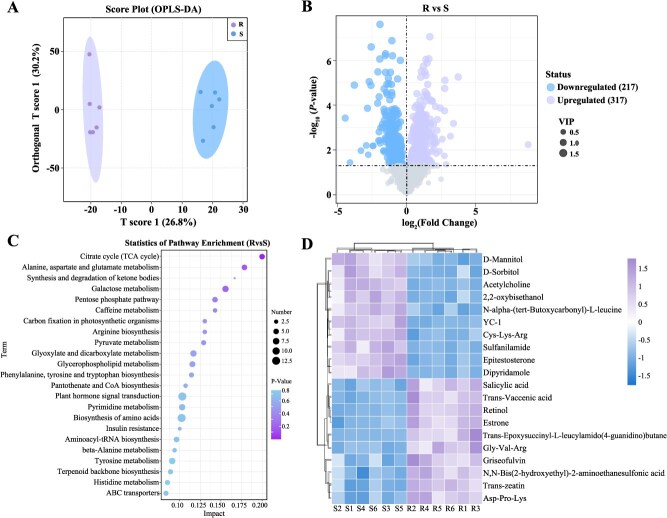
**Variation in leaf metabolites of the resistant and susceptible black currant cultivars to PM infection.** (A) OPLS-DA scores of metabolomes in PM-resistant and susceptible cultivars. (B) Volcano map analysis. (C) KEGG enrichment analysis of DAMs between R and S. (D) The heatmap of metabolites in the top 20 VIP values. The color intensity reflects variations in the relative abundances after normalization and standardization across the samples.

Metabolomics was used to investigate the leaf metabolites in two black currant cultivars (VIP > 1, *P* < 0.05). The Orthogonal partial least square-discriminate analysis (OPLS-DA) demonstrated that the PM-resistant and susceptible cultivars were significantly separated (Fig. 3A). A total of 534 differentially accumulated metabolites (DAMs) were identified, including 317 upregulated and 217 downregulated metabolites in R compared to S (Fig. 3B). In the Kyoto Encyclopedia of Genes and Genomes (KEGG) analysis, these DAMs were enriched in the pathways of ‘Plant hormone signal transduction’ (ath04075), ‘Biosynthesis of amino acids’ (ath01230), and ‘Galactose metabolism’ (ath00052) (Fig. 3C). The expression pattern of metabolites in the top 20 VIP values is shown in Fig. 3D. It was observed that 10 DAMs were significantly upregulated in R compared to S, including SA, *trans*-vaccenic acid, retinol, estrone, *trans*-Epoxysuccinyl-L-leucylamido (4-guanidino) butane, Gly-Val-Arg, griseofulvin, N,N-Bis(2-hydroxyethyl)-2-aminoethanesulfonic acid, *trans*-zeatin, and Asp-Pro-LyS. This indicates that these DAMs were involved in the responses of black currant to PM pathogen in resistant cultivar.

### Microbial diversity and community composition in two cultivars

**Figure 4:**
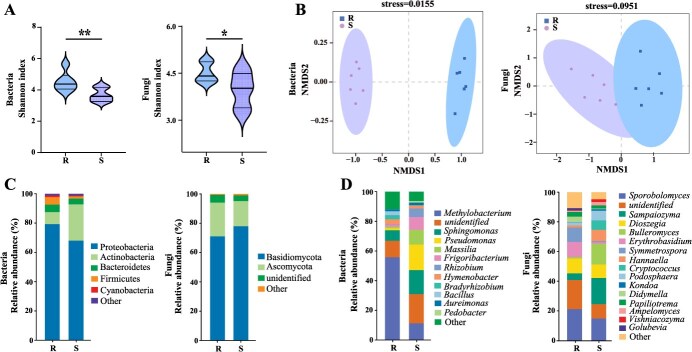
**The composition in phyllosphere microbial community**. (A) Simpson index, statistical significance was denoted by asterisks (^*^P < 0.05, ^**^P <  0.01). (B) NMDS analysis. (C) Relative abundances at the phylum level. (D) Relative abundances at the genus level.

The phyllosphere microbial community diversity and composition in response to PM pathogen were evaluated using high-throughput sequencing. Assessment of bacterial and fungal α-diversity showed that the Shannon index of resistant cultivar increased significantly compared with susceptible cultivar (Fig. 4A (bacteria: *P* < 0.05, fungi: *P* < 0.01)). Furthermore, nonmetric multidimensional scaling analysis (NMDS) of bacteria exhibited apparent differences in phyllosphere microbial community between R and S (stress = 0.0155). Similarly, significant differences in fungal NMDS analysis (stress = 0.0951) were also found between R and S (Fig. 4B). Proteobacteria and Actinobacteria dominated the bacterial communities, while Basidiomycota and Ascomycota dominated the fungal communities in R and S at the phylum level (Fig. 4C). The genera level classification of dominant bacterial and fungal also changed. The relative abundance of the dominant bacterial genus *Methylobacterium* in S decreased significantly compared to R (Fig. 4D). In contrast, the abundance of *Sporobolomyces* in fungal community composition increased in R compared with S (Fig. 4D).

### Microbial community biomarkers and keystone, as well as functional annotation to phyllosphere bacteria

**Figure 5:**
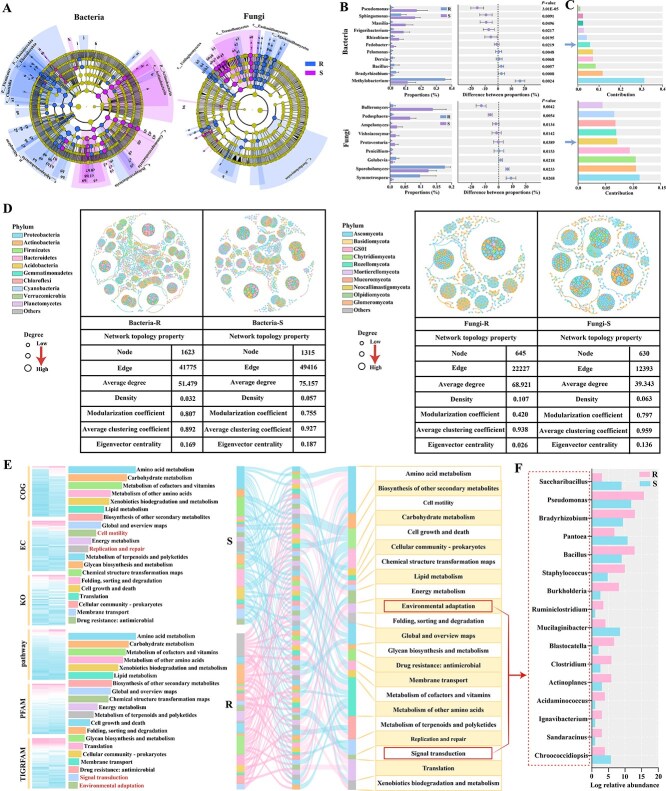
**Prediction of biomarkers and keystone in microbial community, as well as function of phyllosphere bacterial community in R and S.** (A) Bacterial and fungal biomarkers in different cultivars. The circle nodes represent the bacterial and fungal taxa, and the connectivity degree is indicated by the node size. (B) The difference of main bacteria and fungi between R and S according to Welch’s *t*-test (genus level). (C) The SIMPER analysis between R and S. (D) Visual network and topology statistics of bacterial/fungal co-occurrence in R and S. (E) Functional prediction of phyllosphere bacteria based on six databases. COG: Clusters of Orthologous Groups; EC: Enzyme Commission; KO: KEGG Orthology; pathway: KEGG Pathway; PFAM: Protein Family Database; TIGRFAM: TIGR Gene Family Database. **(F)** The relative abundance of 16 bacterial genera with significant differences between R and S.

Linear discriminant biomarker analysis (LefSe) found changes in the dominant populations of bacterial and fungi microbiome between R and S, and discovered some potential biomarkers for resistance to PM infection. The LefSe analysis indicated that the abundance of significantly enriched genera was higher in R compared to S (*P* < 0.05, absolute linear discriminant analysis (LDA) score > 3; Fig. 5A, Fig. S3). The paired *t*-test comparison highlights that *Methylobacterium* and *Pseudomonas* show notable variations between R and S (bacterial microbiome) (Fig. 5B). In the fungal microbiome, the significant differences between R and S were observed in *Symmetrospora* and *Bulleromyces* (Fig. 5B). Furthermore, paired similarity percentage (SIMPER) analysis showed that the primary contributors in bacteria of R versus S were *Methylobacterium*, *Bradyrhizobium*, and *Bacillus*. *Symmetrospora*, *Sporobolomyees*, and *Golubevia* were the top three contributors in fungi (Fig. 5C). The above results showed that the different abundance of primary contributors may lead to different resistance of R and S to PM.

Network topology parameters such as the number of nodes and edges were utilized to assess the complexity of the phyllosphere microbial network. The topological features in bacteria and fungi of the co-occurrence network are shown in Fig. 5D. The average network degrees of the R and S in bacteria were 51.479 and 75.157, respectively. The eigenvector centrality of the bacteria in R was lower than that in S, and the modularity coefficients also exhibited a decline. In the fungal co-occurrence network, the number of edges and average degrees of R were higher than those of S. While it showed that S had a higher modularization coefficient, average clustering coefficient, and eigenvector centrality relative to R (Fig. 5D). In summary, the resistant cultivar exhibited a reduced complexity in the bacterial network, while displaying an increased complexity in the fungal network compared to the susceptible cultivar. The keystones were identified by the highest scores of page ranks or eigen centrality. The bacterial keystones of R were *Roseburia*, *Rhizorhapis*, and *Paenibacillus*, including 10 genera (Page ranks = 0.0008, Eigen centrality = 1), and that of S were *Streptomyces*, *Roseiflexus*, and *Natronincola*, including nine genera (Page ranks = 0.0010, Eigencentrality = 1) (Table S1). In fungal communities, *Sebacina*, *Sarocladium*, and *Rhodotorula*, including 11 keystone genera were identified in R (Page ranks = 0.0016, Eigencentrality = 1). *Apiotrichum*, *Hannaella*, and *Papiliotrema*, including seven keystone genera were identified in S (Page ranks = 0.0019, Eigencentrality = 1) (Table S1).

Functional prediction of phyllosphere bacteria between R and S were analyzed based on six databases. The functional preferences of top 20 revealed that ‘environmental adaptation’ and ‘signal transduction’ were unique to R, ‘cell motility’ and ‘replication and repair’ were unique to S (Fig. 5E, Table S2). The abundance of genera belonging to ‘environmental adaptation’ and ‘signal transduction’ pathways were analyzed, and it was found that 16 bacterial genera showed significant difference in abundance (Fig. 5F, Table S3). We speculated that it is these bacteria that make R resist the invasion of PM and increase disease resistance. Therefore, they were named ‘disease resistance effectors’ of bacteria. More importantly, *Bacillus* and *Burkholderia* were not only ‘disease resistance effectors’, but also bacterial keystone of R.

### Phyllosphere bacterial and fungal correlations analysis

**Figure 6:**
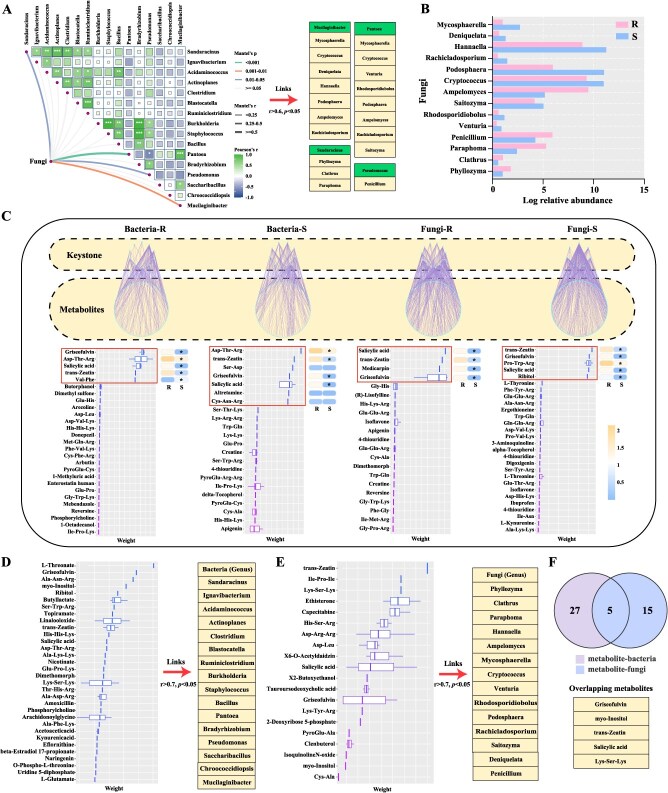
**Metabolome and phyllosphere microbiome interactions**. (A) Mantel tests of the ‘disease resistance effectors’ of bacteria and fungi networks (*r* > 0.6, *P* < 0.05). (B) The relative abundance of core fungal genera between R and S. (C) The microbial community characteristic structure between R and S, and the prediction of weight ranking of high-effects metabolites, as well as the heatmap of distinct metabolites. (D) A Spearman correlation model (*r* > 0.7, *P* < 0.05) between ‘disease resistance effectors’ of bacteria and metabolite, and the metabolite weight ranking. (E) A Spearman correlation model (*r* > 0.7, *P* < 0.05) between core fungal genera and metabolite, and the metabolite weight ranking. (F) A Venn diagram based on the statistics of the frequent occurrence of the same metabolites in the two groups of metabolite–bacteria versus metabolite–fungi.

Mantel test and correlation analysis showed the relationship between bacteria and fungi in the phyllosphere. The ‘disease resistance effectors’ (16 genera) of bacteria were conducted to search for core fungal genera (‘disease resistance effectors’ of fungi) related to PM resistance. There were four bacterial genera significantly related to the fungal networks, including *Mucilaginibacter*, *Sandaracinus*, *Pantoea*, and *Pseudomonas* (Fig. 6A). Furthermore, the four bacterial genera were significantly correlated with 14 genera of fungal networks (*r* > 0.6, *P* < 0.05, Fig. 6B). Next, the relative abundance of 14 core fungal genera under R and S were compared. In these core fungal genera, the relative abundance of *Ampelomyces*, *Penicillium*, *Paraphoma*, *Clathrus*, and *Phyllozyma* in R were significantly higher than S (Analysis of Variance) (Fig. 6B). In particular, *Penicillium* belonging to core fungal genera was also fungal keystone of R.

### Metabolites regulate microbial community variation in two cultivars

Metabolites can affect the microbial community to increase plant resistance. Consequently, the correlation model of the metabolites and bacterial/fungal keystones was employed to investigate primary factors influencing microbial community variations. The keystones and community matrix of bacteria in R and S were closely correlated with several metabolites, including griseofulvin, Asp-Thr-Arg, SA, and *trans*-zeatin (weight and module connectivity were higher) (Fig. 6C). Meanwhile, the expression level of griseofulvin, Asp-Thr-Arg, SA, and *trans*-zeatin metabolites were upregulated in R compared with S. Interestingly, griseofulvin, SA, and *trans*-zeatin metabolites also have a close connection with the fungal keystones in R and S (Fig. 6C). In addition, a Spearman correlation model and Mantel test between ‘disease resistance effectors’ of bacteria/fungi and metabolite weight ranking were also analyzed. (Fig. 6D and E). There were 32 high-weight metabolites related to bacterial ‘disease resistance effectors’, and 20 high-effects metabolites were associated with ‘disease resistance effectors’ in fungi (*r* > 0.7, *P* <  0.05). Five metabolites had binary overlap (griseofulvin, myo-Inositol, *trans*-zeatin, SA, Lys-Ser-Lys), indicating that metabolites affected both bacterial and fungal communities to resist pathogen invasion, and there were complex interactions among them (Fig. 6F). Based on the above results, SA, *trans*-zeatin, and griseofulvin were considered as key metabolites in black currant response to PM infection.

**Figure 7:**
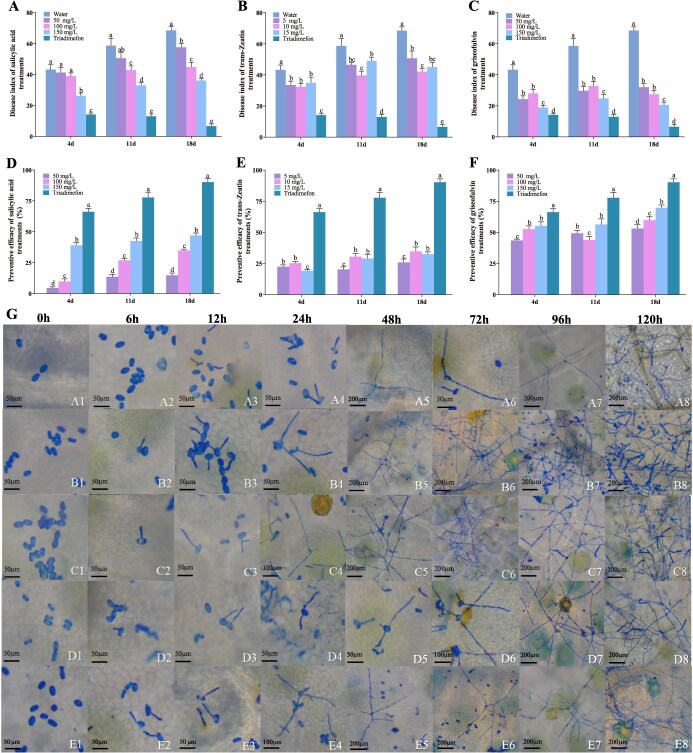
**Validation of three key metabolites acting on PM pathogen suppression**. (A–C) The disease indexes of plants treated with different concentrations of SA, *trans*-zeatin. and griseofulvin, respectively. (D–F) The preventive efficacy of plants treated with different concentrations of SA, *trans*-zeatin, and griseofulvin, respectively. (G) Spore growth characters with spraying water and different concentrations of griseofulvin at 0, 6, 12, 24, 48, 72, 96, and 120 h after PM pathogen inoculation. A1–A8: Spray water on resistant cultivar; B1–B8: Spray water on susceptible cultivar; C1–C8: Spray 50 mg/l griseofulvin on susceptible cultivar; D1–D8: Spray 100 mg/l griseofulvin on susceptible cultivar; E1–E8: Spray 150 mg/l griseofulvin on susceptible cultivar.

### Effects of three key metabolites on powdery mildew pathogen

To investigate the potential roles of SA, *trans*-zeatin, and griseofulvin in resisting PM, the ability of three key metabolites to reduce the occurrence of PM in susceptible cultivar was tested. Their impacts on disease incidence were monitored and recorded over a 21-day timeframe. Phenotypic comparison of spraying three metabolites was observed. (Fig. S4). The white powdery fungal colonies on the leaves of ‘Bright Leaf’ treated with griseofulvin at different concentrations were fewer than those treated with water (Fig. S4). The disease indexes of plants treated with SA, *trans*-zeatin, and griseofulvin were all lower than that of the spraying water group and higher than that of triadimefon treatment at 4, 11, and 18 days (Fig. 7A–C). At the same time, we observed that preventive efficacy increased significantly in three key metabolite treatments, relative to the water group (*P* <0 .05, Fig. 7D–F). The results of disease indexes and preventive efficacy demonstrated that 150 mg/l SA, 10 mg/l *trans*-zeatin, and 150 mg/l griseofulvin exhibited more excellent control for PM in different concentration groups. However, compared with spraying 150 mg/l SA and 10 mg/l *trans*-zeatin, the preventive efficacy of 150 mg/l griseofulvin was elevated by 18.87% and 30.10% at 18 days, respectively (Fig. 7A–C). In addition, compared with the other two metabolites, griseofulvin with different concentrations has better performance in responding to PM infection. The plant preventive efficacy of spraying three different concentrations of griseofulvin at 18 days significantly increased by 24.25%, 33.76%, and 48.80% compared to water, respectively (*P* <  0.05, Fig. 7C). After spraying different metabolites for three times, the best preventive efficacy was 69.77% by 150 mg/l griseofulvin. Overall, the results showed that 150 mg/l griseofulvin had better control effect on PM than different concentrations of SA and *trans*-zeatin.

The influence of three key metabolites on the PM infection process was observed by Coomassie brilliant blue staining. When spores were inoculated on the leaves of PM-resistant cultivar and sprayed with water, the spores could not complete the asexual growth cycle within 120 h (Fig. 7G, A1–A8). Spores could complete their asexual growth cycle within 120 h after inoculation and spraying water on PM-susceptible cultivar (Fig. 7G, B1–B8). When 50 and 100 mg/l griseofulvin were applied to susceptible cultivar, the asexual growth cycle of spores was completed at 96 and 120 h, respectively (Fig. 7G, C1–C8 and D1–D8). However, the application of 150 mg/l griseofulvin led to spores failing to complete their asexual growth cycle within 120 h (Fig. 7G, E1~E8). It was found that the application of 5 mg/l *trans*-zeatin makes spores complete their asexual growth cycle within 120 h (Fig. S5, F1–F8). When the concentrations of *trans*-zeatin were 10 and 15 mg/l, the spore did not complete their asexual growth cycle within 120 h (Fig. S5, G1–G8 and H1–H8). The spores could complete their asexual growth cycle within 96 h after spraying 50 mg/l SA (Fig. S5, I1–I8). The use of 100 and 150 mg/l SA treatments resulted in spores completing their asexual growth cycle within 120 h (Fig. S5, J1–J8 and K1–K8). These findings indicate that the application with griseofulvin (150 mg/l) and *trans*-zeatin (10 and 15 mg/l) shows decelerating the infection process of PM pathogen, compared to spraying water on susceptible cultivar.

## Discussion

Yield losses and quality degradation caused by PM pathogens pose a major threat to black currant. Understanding the resistance mechanism in the face of PM is crucial for plants breeding and agricultural practices. Growing evidence suggests that plants experiencing pathogen infection utilize a series of chemical signals to attract health-promoting microbes and enhance their capabilities, which is known as the plant-mediated ‘cry for help’ [34–36]. Most studies have concentrated on the response of rhizosphere microorganisms to pathogens in the underground environment [8, 13, 37], while little is known about the microorganisms in the phyllosphere (the aboveground part). In the current research, PM fungus species were identified and leaf structure differences were observed in resistant and susceptible black currant cultivars. Based on high-throughput sequencing and metabolomic analyses, we further explored the differences of key metabolite and phyllosphere microbial communities in two cultivars upon PM pathogen infection. More importantly, the changes in host metabolites regulating microbial community to resist fungal pathogens was elucidated in plant phyllosphere.

Identifying PM fungus species is essential for effective management of this disease. Many species of PM fungi can be distinguished by morphological traits and molecular sequence analysis [38, 39]. Based on the results of the conidiophore morphology and phylogenetic tree further confirmed that *P. mors-uvae* was PM pathogen in black currant (Fig. 1A and B). Recognizing the pathogen is merely the first step; understanding plant’s defense mechanisms against pathogen invasion is equally critical. First, inherent physical structures provide base defense against fungal pathogens [40]. In the complex process of interaction between host and pathogen, plants can form physical barriers through the changes of epidermis waxy layer and cuticle to resist the fungal pathogen invasion [41, 42]. The thickness of cuticle, sponge tissue, and fence tissue of resistant cultivar were significantly higher than that of the susceptible cultivar (Fig. 2C, *P* < 0.05). In addition, stomata are the gateway for gas exchange between plants and atmosphere, and it is also an important channel for pathogens to infect plants [43]. The reduced stomatal number and leaf area ratio of stomata were observed in resistant cultivar of black currant (Fig. 2B and C). These results indicate that increased leaf tissue thickness and reduced stomatal number can resist pathogen invasion, thereby improving PM resistance. Second, plants can resist pathogens through secretion of various chemical molecules, including endogenous phytohormones, signaling molecules, and secondary metabolites [44]. In this study, a total of 534 DAMs were identified, and these DAMs were enriched in ‘Plant hormone signal transduction’ pathway (Fig. 3B and C). More importantly, 10 DAMs were significantly upregulated in resistant cultivar compared to susceptible cultivar (Fig. 3D). The resistant cultivar exhibited greater resistance to PM than the susceptible cultivar, leading us to speculate that this difference may be attributed to variations in key differential metabolites that contribute to PM resistance.

Plant cultivars differing in resistance against pathogens can modulate the characteristics and functional attributes of microorganisms [45, 46]. Previous studies have found that the microbial communities of fusarium wilt-resistant and susceptible cabbage varieties were markedly distinct, and the introduction of the pathogen elicited significant changes in their microbial networks [46]. Studies on microbial communities of corn stalk rot-resistant and susceptible maize varieties also showed that resistant plants controlled the assembly of beneficial microbial communities [47]. In line with that, we found the composition of microbial communities was significantly different in two black currant cultivars (Fig. 4). The higher Shannon index of bacteria and fungi were observed in resistant cultivar (Fig. 4A). This high α-diversity demonstrated some level of resilience to PM pathogen attacks, maintaining microbial community stability. The beta-diversity analysis also showed differences in the composition of resistant and susceptible cultivars (Fig. 4B). The results demonstrated that resistant cultivar has the strong ability to select their microbial composition, providing advantages in dealing with pathogen invasion.

**Figure 8:**
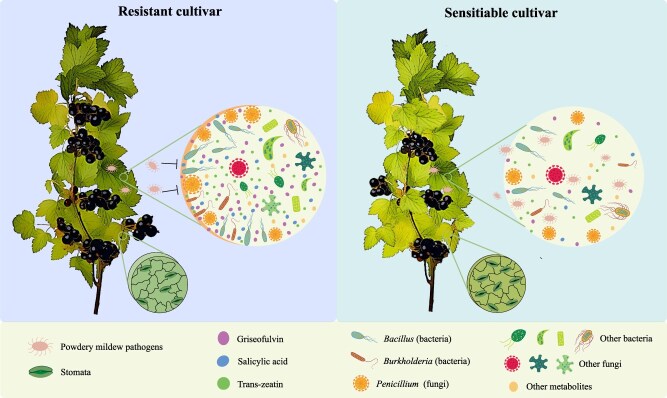
The mechanism of resistant cultivar enhances resistance to PM pathogens in the black currant phyllosphere.

Network analysis facilitates a deeper understanding of the interactions and identifies keystone species that make the largest impacts on microbial communities [48, 49]. This study found much greater numbers of nodes and edges in the bacterial network than the fungal network (Fig. 5D), suggesting phyllosphere bacteria were more active in response to PM pathogen attacks. Additionally, the resistant cultivar demonstrated a lower complexity in the bacterial network, whereas it showed a higher complexity in the fungal network compared with the susceptible cultivar. The findings revealed that the defense mechanisms in resistant cultivar may suppress specific bacterial populations, resulting in a simplified bacterial network. Conversely, the increase of fungal network complexity in resistant cultivar implied a greater reliance on beneficial fungi for nutrient acquisition and disease resistance, which promotes a more intricate and diverse fungal community [50]. Through the keystone identification and function prediction of bacteria, our results showed *Bacillus* and *Burkholderia*, enriched in the resistant cultivar, were identified as the keystone species and ‘disease resistance effectors’ in R (Fig. 5E, Table S1). It has been reported that *Bacillus* species were recognized for antagonistic properties against a variety of plant pathogens [51]. *Burkholderia* can establish beneficial associations with plant roots, improving nutrient uptake. Some studies shown that *Burkholderia* induced systemic acquired resistance in plants, which enables plants to cope with subsequent pathogen attacks more effectively [52]. Based on the topological features and correlation analysis, *Penicillium* was identified as the fungal keystone of R and core fungal genera related to PM resistance (Fig. 6A, Table S1). Certain species of *Penicillium* can produce bioactive compounds that have antifungal and antibacterial properties, thereby protecting plants from various pathogens [53]. Taken together, *Bacillus*, *Burkholderia*, and *Penicillium* were beneficial microbes to promoting PM resistance.

Host plants can modulate microbial communities via secreting various chemical molecules, including endogenous plant hormones, signaling molecules, and secondary metabolites [54–56]. In this study, weighted metabolite–microbial coexpression network analysis revealed that griseofulvin, SA, and *trans*-zeatin were significantly correlated with the microbial keystones and ‘disease resistance effectors’ (Fig. 6C–E). The resistant cultivar may alleviate pathogen attacks by promoting the release of these metabolites, which leads to enrichment of beneficial microorganisms. Our results were consistent with astaxanthin inhibiting the growth and pathogenicity of *Ralstonia solanacearum* through recruiting beneficial microorganisms in resistant tomato cultivars [57]. Similarly, with the expansion of rust lesions, metabolites such as terpenoids, flavonoids, and lignin precursors had a positive regulatory effect on microbial taxa in crabapple species ‘Kelsey’ [24]. Our research has also shown that spraying different concentrations of SA, *trans*-zeatin, and griseofulvin could improve the resistance to PM, and 150 mg/l griseofulvin had better control efficiency by inhibiting fungal cell mitosis and growth, decelerating the asexual growth cycle of spores (Fig. 7G). Previous studies have reported that griseofulvin showed high antifungal activity *in vivo* and *in vitro*, and effectively inhibited the development of barley PM, rice blast, rice sheath blight, and wheat leaf rust [58]. Griseofulvin targets *Didymella segeticola* beta-Tubulin to disturb the chromosomal separation and fungal mitosis, thereby inhibiting the growth of tea leaf spot hyphae [59]. Based on these findings, it is suggested that the resistant cultivar enhance resistance to PM pathogens through multilevel defense responses (Fig. 8). (i) The resistant cultivar formed inherent physical structures to provide base defense, including fewer stomata and increased leaf tissue thickness. (ii) Resistant cultivar showed a higher diversity of microbial communities and an increased complexity of the fungal co-occurrence network. (iii) SA, *trans*-zeatin, and griseofulvin were more abundant in resistant cultivar, positively influencing the enrichment of beneficial microorganisms in the phyllosphere.

## Conclusion

In this work, the differences of inherent physical structures, microbial communities, and metabolites associated with PM-resistant and susceptible black currant cultivars were investigated. First, the increased leaf tissue thickness and reduced stomatal number of resistant cultivars provided base defense against PM pathogens. Second, *Bacillus*, *Burkholderia* (bacteria), and *Penicillium* (fungi) species were core microbiota of resistant cultivar that induce the host to improve PM resistance. Last, the changes of important metabolites, particularly griseofulvin, SA, and *trans*-zeatin, exhibited a significant correlation with the keystone species and ‘disease resistance effectors’ of both bacteria and fungi, thereby mediating variations in microbial community. A total of 150 mg/l griseofulvin had better control effect on pathogen invasion. In summary, PM-resistant black currant cultivar possesses multilayered chemical and physical defenses in the phyllosphere. These findings enhance our understanding for the roles of metabolites and microbiotas as the core factors in responses to PM challenge and lay the groundwork for assisting agricultural practices to improve PM resistance. Future research will further elucidate the effects of core bacterial and fungal isolates on PM disease suppression and the potential mechanism between microorganisms and PM resistance. By integrating these insights into agricultural practices, we can promote healthier crops, dependence on chemical pesticides, and finally achieve more sustainable disease management strategies.

## Materials and methods

### Plant materials and cultivation

Based on the investigation of PM at the black currant Germplasm Nursery of Northeast Agricultural University in Harbin, China (125°38′ E, 45°45’N), black currant resistant cultivar ‘16A’ and susceptible cultivar ‘Bright leaf’ were used in this experiment. The middle and upper parts of shoots from two cultivars were used as cuttings. Each cutting was ~2 to 3 knots, and 1–2 leaves were reserved. The leaves on the cuttings were trimmed to half the original size, soaked in rooting powder solution for 2 min, inserted the treated cutting into the greenhouse to the depth of the petiole base, sprayed with water on the nursery after all the cuttings are inserted, and then watered once a day. The growth conditions in the greenhouse were average temperatures of 24°C/18°C (day/night), 60% relative humidity, and the daily illumination of 12 h (700 mmol·m^−2^·s^−1^).

### Pathogen infection and sample collection

When ‘16A’ and ‘Bright leaf’ materials grow to ~20 cm, the separated and purified fresh PM spores were evenly shaken off to the leaves. The six cutting seedlings with the same growth potential were selected from two cultivars, and one leaf was collected from each seedling at the same position after 15 days of inoculation. Some leaves were immediately stored at −80°C for RNA extraction and metabolite analysis. Another part of the collected leaves was placed in a 50 ml centrifuge tube, and 30 ml PBS solution was added to the centrifuge tube. Shake the blades in a shaker (THZ-100, China) at 25°C and 150 rpm for 30 min, and collect the cleaning solution. Repeat the above steps three times. Centrifuge the cleaning solution at 4°C and 12 000 rpm for 15 min, discard the supernatant, and collect the bacterial liquid. The obtained plaque was eluted with sterile water and collected into a 1.5 ml centrifuge tube for the extraction of microbial DNA.

### Powdery mildew of black currant pathogen identification

#### Isolation, purification, and morphological identification

The fresh leaves of black currant (‘Bright leaf’) with PM were collected from the Germplasm Nursery. The surfaces of the diseased leaves were rinsed with sterile distilled water to remove the old spores. Then the leaves were laid flat on potato dextrose agar (PDA), and we waited until they produced a large number of fresh conidia before removing them. The conidia were shaken off in Tween 20 solution to make a spore suspension of ~1 × 10^5^ ml^−1^, which was sprayed on the leaves of ‘Bright leaf’. The plants were placed in the greenhouse for cultivation, and the sterile water solution with Tween 20 was used as controls. After four rounds of isolation and purification, the PM spores were placed on the glass slide and observed using a 10 × 40× optical microscope (MED D45T, Spain).

#### Molecular identification

Total genomic DNA was isolated from the pathogen of black currant PM refers to the improved Chelex-100 method [60]. The ITS sequence was amplified using the primers ITS4/ITS5 (Table S4). The reaction procedure was 94°C for 2 min, 35 cycles (94°C for 30 s, 50°C for 30 s, 72°C for 45 s), 72°C for 10 min, and 4°C termination. PCR products were purified and ligated to vector. It was transformed into *Escherichia coli*, and the positive strain was sequenced.

#### Pathogenicity detection

According to Koch’s Rule, the pathogenicity of PM pathogen was determined by reconnection test of isolated fungi. The isolated and purified pathogenic fungi were prepared into spore suspension of ~1 × 10^5^ ml^−1^, and 10 leaves of ‘Bright leaf’ were inoculated using the spray method. Mock-inoculated leaves (spray water) were used as controls. After 10 days, ‘Bright leaf’ and mock-inoculated leaves were observed for disease situation. The pathogen was isolated from the leaves again, and the similarities and differences between it and the inoculated pathogen were observed.

### Phylogenetic analyses and sequence alignment

The sequence of PM in black currant was uploaded, and the most similar sequences were searched by using BLAST in NCBI database. These sequences were aligned for constructing the phylogenetic tree using Clustal W. Based on tandem sequences of the ITS, the phylogenetic tree was generated using the maximum likelihood (ML) method in MEGA v.11.0 software [61]. Bootstrap analysis was made using 1000 replications. Phylogenetic tree beautification was used as IToL.v6 software(https://itol.embl.de/).

### Observation of leaf structure of PM-resistant and susceptible cultivars

#### Lower epidermal stomata of leaves

After washing with distilled water, the fresh leaves of ‘16A’ and ‘Bright leaf’ were cut into 0.5-cm segments. We then immediately tore off the lower epidermis and placed them in a drop of water on the slide. Cover glass was covered to make temporary slicing. The samples were placed on an optical microscope (Nikon E200MV, Japan) to observe, and leaf stomatal number were recorded (four samples were selected for each cultivar).

#### Leaf cross-section

The ‘16A’ and ‘Bright leaf’ fresh leaves were put on the smooth glass. Along the direction perpendicular to the main vein, the leaves were quickly cut by two blades side by side, and then put into a petri dish filled with water. The thinnest slice was selected and put in the water drop on the glass slide, and covered with a cover glass to make the slicing. The leaves cross-sections were observed using optical microscope and the values of leaf thickness, cuticle thickness, sponge tissue thickness, and fence tissue thickness were measured and recorded (four samples were selected for each cultivar).

### Extraction, detection, and analysis of metabolites

Fifty milligrams of leaf samples from ‘16A’ and ‘Bright leaf’ were put in liquid nitrogen and quickly ground into powder, respectively. One hundred microliters of homogenized samples were put into a 2 ml microcentrifuge tube and mixed with 400 μl of extraction solution (methanol: acetonitrile = 1:1), and vortexed for 60 s. The solution was subjected to ultrasonic treatment for 10 min at 20°C and centrifuged for 15 min at 13 000 rpm at 4°C. Approximately 350 μl of supernatant was transferred to a 1.5 ml microcentrifuge tube, and the sample was dried completely in a vacuum concentrator. Subsequently, the sample was mixed with 150 μl of extraction solution (methanol: acetonitrile = 1:1) to resolve. The samples were vortexed for 30 s, subjected to ultrasonic treatment for 10 min, and then centrifuged for 15 min at 13 000 rpm at 4°C. The supernatant was used as the solution to be tested.

The LC–MS was performed using the ultrahigh-performance liquid tandem chromatography quadrupole time of flight mass spectrometry. The raw data were fed to R v4.3.1 *metaX* for OPLS-DA. The DAMs were identified by a combination of variable importance in projection (VIP) > 1 and *P* < .05.

### DNA extraction, 16S rRNA, and ITS amplicon sequencing

Total DNA of phyllosphere microorganisms resistant and susceptible cultivars was extracted by PowerSoil DNA Isolation Kit (Mobio, USA). The concentration of the DNA samples was determined using NanoDrop ND-8000 spectrophotometer (NanoDrop, USA). The V3–V4 region of 16S rRNA primers were 338F and 806R. The ITS primers were ITS1F and ITS2R (the primer sequences were shown in Table S5). The PCR products were detected by 1% agarose gel electrophoresis, and purified by Agencourt AMPure XP nucleic acid purification kit (Beckman Coulter, Germany). The purified PCR products were sent to Beijing Ovison Gene Technology Co., Ltd. for high-throughput sequencing. The constructed library was sequenced using the Illumina Miseq PE300 platform (Illumina, USA). The raw sequencing data were deposited at the NCBI Sequence Read Archive (SRA) database (accession number: PRJNA1186754).

### Microbial informatics analysis

The raw sequencing data was processed with Trimmomatic 0.33 to ensure nucleotide quality. Sequences with a similarity of ≥97% were clustered into operational taxonomic units (OTUs) using QIIME v1.8.0 (http://qiime.org/) [62]. Alpha and beta diversity indices were calculated with QIIME v1.8.0. The NMDS graphs were plotted using vegan and *ggplot2* in R v4.3.1 software. The abundances of bacterial and fungal taxa at phylum and genus levels were statistically compared by MetaStat. LEfSe analysis was performed using LEfSe software and applying a threshold for the LDA score >3. Differences of the MetaStat analysis between the two comparison groups were tested in R v4.3.1, and obtain the *P*-values. Species with *P* < 0.05 were identified as significantly different species. Significant species differences at various taxonomic levels were detected using the *t*-test. Microbial co-occurrence networks were analyzed and visualized using R v4.3.1 and Gpehi v 0.10.1. Topology parameters were calculated using Gpehi v 0.10.1. The PICRUSt 2 was used to construct the evolutionary tree by aligning the characteristic sequence (16S rRNA) and the reference sequence of microbial genome database. Then, the ‘nearest species’ of the characteristic sequence were found. According to the gene function spectra of known species, gene functional spectrum of other unknown species was predicted. Finally, the gene function prediction spectrum of the whole pedigree of bacterial domain was constructed [63]. Mulberry map was created with R v4.3.1 *ggalluvial* package, and data was visualized with *ggplot2*.

### Correlation analysis of microbial and metabolites

Mantel test used the R *linkET* package and the threshold was set to *r* > 0.6 and *P* <  0.05. According to the fungus matrix and bacterial matrix, the distance matrix of two kinds of data was transformed by Vegdist function. Then, the Spearman correlation of two kinds of matrices was analyzed by Mantel function to obtain *r* and *P*-values [64]. Spearman correlation coefficient between keystones and metabolites was calculated by the Corr. test function of R v4.3.1 *Psych* package, and their significance was tested. The correlation between characteristic metabolite and microbial groups was shown by Cytoscape v.3.10.0. The highly correlated metabolites were ranked from high to low by R v4.3.1 *ggpubr* package (*r* > 0.6, *P* < 0.05), and visualization was conducted using the R v4.3.1 *Pheatmap* function. Spearman and Mantel tests were used to show the corresponding relationship between metabolites–bacteria and metabolites–fungi, respectively. In order to improve the accuracy, the threshold was set to *r* > 0.7, *P* <  0.05.

### Validation of three key metabolites in response to powdery mildew in black currant

Three different concentrations of three key metabolites were sprayed on leaves of the susceptible cultivar ‘Bright leaf’: (1) 50, 100, and 150 mg/l of SA; (2) 5, 10, and 15 mg/l of *trans*-zeatin; and (3) 50, 100, and 150 mg/l of griseofulvin. Water and triadimefon (common pesticides for PM of black currant) were used as controls. Eleven treatments were set up to examine the effects of differential metabolites on PM. Treatments was conducted using 33 susceptible cultivar ‘Bright leaf’ of the black currant Germplasm Nursery in three replicates (each tree was treated as one). Fifty leaves were randomly selected from each tree to investigate the incidence level of PM. The plants were sprayed with three metabolites, water, and 500 times of triadimefon once every 7 days, for a total of three times. The disease index and preventive efficacy were investigated before spraying, on the fourth day after the first spraying (4 days), on the fourth day after the second spraying (11 days), and on the fourth day after the third spraying (18 days), respectively.

Disease index = 100 × ∑ (Disease level value × Leaf number at each level)/(Total leaf number × the highest level) (Classification levels of PM disease of black currant, Table S6).

Preventive efficacy (%) = (control disease index-treatment disease index)/control disease index × 100%.

The leaves of the susceptible cultivar ‘Bright leaf’ cultivated in greenhouse were sprayed with SA (50, 100, and 150 mg/l), *trans*-zeatin (5, 10, and 15 mg/l), and griseofulvin (50, 100, and 150), respectively. Each solution was set with three concentrations, and each treatment was repeated for three times. Water was used as a control, and then plants are infected by PM pathogen. The inoculation method was the same as that in Sections 5.2. Samples were collected at 0, 6, 12, 24, 48, 72, 96, and 120 h after spraying three metabolites and water, and three samples were taken for each treatment. According to the method [65], the leaves were decolorized transparently, dyed with Coomassie brilliant blue for 150 s, and then the growth of spores was observed by Olympus optical microscope. In addition, the spore germination rate was observed and recorded after spraying metabolites and water for 12 h.

### Statistical analyses

The GraphPad Prism 8 (GraphPad Software, USA) and R v4.3.1 were used for plotting. One-way analyses of variance and Tukey’s method were performed using SPSS 22.0 (SPSS Inc., USA) to estimate the differences leaf structure, alpha diversity, disease indexes, and preventive efficacy.

## Supplementary Material

Web_Material_uhaf092

## Data Availability

All relevant data in this study are provided in the article and its supplementary material.

## References

[1] Woznicki TL, Heide OM, Sønstebyc A. et al. Effects of controlled post-flowering temperature and daylength on chemical composition of four black currant (*Ribes nigrum* L.) cultivars of contrasting origin. Sci Hortic. 2015;197:627–36

[2] Qin D, Wang H, Zhang C. et al. Effects of GA3 and ABA on the respiratory pathways during the secondary bud burst in black currants. J For Res. 2017;28:705–12

[3] Qin D, Zhao L, Gary G. et al. Effects of fruit thinning on ascorbate–glutathione cycle metabolism in black currants (*Ribes nigrum* L.). J For Res. 2017;28:903–8

[4] Li W, Qin D, Ma R. et al. Comparative evaluation of physiological and molecular responses of black currant varieties to powdery mildew infection. Front Plant Sci. 2024;15:1445839 39354936 10.3389/fpls.2024.1445839 PMC11442278

[5] Kampuss K, Strautina S, Kampuse S. Red and white currant genetic resources in Latvia. Acta Hortic. 2007;760:397–403

[6] Vandenkoornhuyse P, Quaiser A, Duhamel M. et al. The importance of the microbiome of the plant holobiont. New Phytol. 2015;206:1196–206 25655016 10.1111/nph.13312

[7] Zhang J, Liu Y, Zhang N. et al. *NRT1.1B* is associated with root microbiota composition and nitrogen use in field-grown rice. Nat Biotechnol. 2019;37:676–84 31036930 10.1038/s41587-019-0104-4

[8] Kwak MJ, Kong HG, Choi K. et al. Rhizosphere microbiome structure alters to enable wilt resistance in tomato. Nat Biotechnol. 2018;36:1100–9 10.1038/nbt.4232 30295674

[9] Lau JA, Lennon JT. Rapid responses of soil microorganisms improve plant fitness in novel environment. Proc Natl Acad Sci USA. 2021;109:14058–62 10.1073/pnas.1202319109 PMC3435152 22891306

[10] Wurst S, van Beersum S, Wagenaar R. et al. Plant defence against nematodes is not mediated by changes in the soil microbial community. Funct Ecol. 2009;23:488–95

[11] Bai B, Liu W, Qiu X. et al. The root microbiome: community assembly and its contributions to plant fitness. J Integr Plant Biol. 2022;64:230–43 35029016 10.1111/jipb.13226

[12] Gao M, Xiong C, Gao C. et al. Disease-induced changes in plant microbiome assembly and functional adaptation. Microbiome. 2021;9:1–18 34526096 10.1186/s40168-021-01138-2 PMC8444440

[13] Yin C, Casa Vargas JM, Schlatter DC. et al. Rhizosphere community selection reveals bacteria associated with reduced root disease. Microbiome. 2021;9:86 33836842 10.1186/s40168-020-00997-5 PMC8035742

[14] Seybold H, Demetrowitsch TJ, Hassani MA. et al. A fungal pathogen induces systemic susceptibility and systemic shifts in wheat metabolome and microbiome composition. Nat Commun. 2020;11:1910 32313046 10.1038/s41467-020-15633-x PMC7171108

[15] Wen T, Xie P, Liu H. et al. Tapping the rhizosphere metabolites for the prebiotic control of soil-borne bacterial wilt disease. Nat Commun. 2023;14:4497 37495619 10.1038/s41467-023-40184-2 PMC10372070

[16] Trivedi P, Leach JE, Tringe SG. et al. Plant–microbiome interactions: from community assembly to plant health. Nat Rev Microbiol. 2020;18:607–21 32788714 10.1038/s41579-020-0412-1

[17] Oyserman BO, Flores SS, Griffioen T. et al. Disentangling the genetic basis of rhizosphere microbiome assembly in tomato. Nat Commun. 2022;13:3228 35710629 10.1038/s41467-022-30849-9 PMC9203511

[18] Jin X, Jia H, Ran L. et al. Fusaric acid mediates the assembly of disease-suppressive rhizosphere microbiota via induced shifts in plant root exudates. Nat Commun. 2024;15:5125 38879580 10.1038/s41467-024-49218-9 PMC11180119

[19] Mendes LW, Mendes R, Raaijmakers JM. et al. Breeding for soil-borne pathogen resistance impacts active rhizosphere microbiome of common bean. The ISME Journal. 2018;12:3038–42 30018368 10.1038/s41396-018-0234-6 PMC6246553

[20] El-Hadidy AM, El-Ati AA. Efficiency of effective microorganisms (EM) to induce resistance against chocolate spot disease and enhance productivity of Faba bean under reclaimed soil conditions. Egypt J Phytopathol. 2014;42:117–42

[21] Jacoby RP, Koprivova A, Kopriva S. Pinpointing secondary metabolites that shape the composition and function of the plant microbiome. J Exp Bot. 2021;72:57–69 32995888 10.1093/jxb/eraa424 PMC7816845

[22] Berendsen RL, Pieterse CMJ, Bakker PAHM. The rhizosphere microbiome and plant health. Trends Plant Sci. 2012;17:478–86 22564542 10.1016/j.tplants.2012.04.001

[23] Koski TM, Zhang B, Mogouong J. et al. Distinct metabolites affect the phloem fungal communities in ash trees (*Fraxinus* spp.) native and nonnative to the highly invasive emerald ash borer (*AGRILUS PLANIPENNIS*). Plant Cell Environ. 2024;47:4116–34 38922989 10.1111/pce.14996

[24] Zhang Y, Cao B, Pan Y. et al. Metabolite-mediated responses of phyllosphere microbiota to rust infection in two *Malus* species. Microbiology Spectrum. 2023;11:e03831–22 36916990 10.1128/spectrum.03831-22 PMC10101083

[25] Kudjordjie EN, Sapkota R, Steffensen SK. et al. Maize synthesized benzoxazinoids affect the host associated microbiome. Microbiome. 2019;7:59 30975184 10.1186/s40168-019-0677-7 PMC6460791

[26] Hassan S, Mathesius U. The role of flavonoids in root-rhizosphere signalling: opportunities and challenges for improving plant-microbe interactions. J Exp Bot. 2012;63:3429–44 22213816 10.1093/jxb/err430

[27] Stringlis IA, Yu K, Feussner K. et al. MYB72-dependent coumarin exudation shapes root microbiome assembly to promote plant health. Proc Natl Acad Sci USA. 2018;115:5213–22 10.1073/pnas.1722335115 PMC5984513 29686086

[28] Lebeis SL, Paredes SH, Lundberg DS. et al. Salicylic acid modulates colonization of the root microbiome by specific bacterial taxa. Science. 2015;349:860–4 26184915 10.1126/science.aaa8764

[29] Cowley T, Walters DR. Polyamine metabolism in barley reacting hypersensitively to the powdery mildew fungus *Blumeria graminis* f. sp. *hordei*. Plant Cell Environ. 2002;25:461–8

[30] Pociecha E, Janeczko Z, Janeczko A. Resveratrol stimulates phenolic metabolism and PSII efficiency in wheat infected with powdery mildew. J Plant Interact. 2014;9:494–503

[31] Zhou M, Wang H, Yu X. et al. *VviWRKY10* and *VviWRKY30* co-regulate powdery mildew resistance through modulating SA and ET-based defenses in grapevine. Plant Physiol. 2023;195:446–61 10.1093/plphys/kiae080 38366578

[32] Yang F, Wu C, Zhu G. et al. An integrated transcriptomic and metabolomic analysis for changes in rose plant induced by rose powdery mildew and exogenous salicylic acid. Genomics. 2022;114:110516 36306956 10.1016/j.ygeno.2022.110516

[33] Zhang Z, Jiang C, Chen C. et al. *VvWRKY5* enhances white rot resistance in grape by promoting the jasmonic acid pathway. Horticulture Research. 2023;10:uhad172 37841502 10.1093/hr/uhad172 PMC10569242

[34] Rolfe SA, Griffiths J, Ton J. Crying out for help with root exudates: adaptive mechanisms by which stressed plants assemble health-promoting soil microbiomes. Curr Opin Microbiol. 2019;49:73–82 31731229 10.1016/j.mib.2019.10.003

[35] Rizaludin MS, Stopnisek N, Raaijmakers JM. et al. The chemistry of stress: understanding the “cry for help” of plant roots. Meta. 2021;11:357 10.3390/metabo11060357 PMC8228326 34199628

[36] Wang Z, Song Y. Toward understanding the genetic bases underlying plant-mediated “cry for help” to the microbiota. iMeta. 2022;1:e8 38867725 10.1002/imt2.8 PMC10989820

[37] Carrión VJ, Perez-Jaramillo J, Cordovez V. et al. Pathogen-induced activation of disease-suppressive functions in the endophytic root microbiome. Science. 2019;366:606–12 31672892 10.1126/science.aaw9285

[38] Zhang J, Bai L. Morphological and molecular phylogenetic identification of powdery mildew pathogen in *Hylotelephium erythrostictum*. Molecular Pathogens. 2022;13:1–5

[39] Sun X, Ding W, Jiang Y. et al. Morphology, photosynthetic and molecular mechanisms associated with powdery mildew resistance in Kentucky bluegrass. Physiol Plant. 2024;176:e14186 38351885 10.1111/ppl.14186

[40] Ebrahim S, Usha K, Singh B. Plant architectural traits and their role in defense mechanism against malformation in mango (*Mangifera Indica* L.). Sci Hortic. 2012;139:25–31

[41] Jiang Y, Peng Y, Hou G. et al. A high epicuticular wax strawberry mutant reveals enhanced resistance to *Tetranychus urticae* Koch and *Botrytis cinerea*. Sci Hortic. 2024;324:112636

[42] Xu X, Chen Y, Li B. et al. Molecular mechanisms underlying multi-level defense responses of horticultural crops to fungal pathogens. *Horticulture*. Research. 2022;9:uhac066 10.1093/hr/uhac066 PMC9113409 35591926

[43] Zhang D, Tian C, Yin K. et al. Postinvasive bacterial resistance conferred by open stomata in rice. Mol Plant-Microbe Interact. 2019;32:255–66 30124364 10.1094/MPMI-06-18-0162-R

[44] Wang L, Xia Y, Hou Y. Candy or poison: plant metabolites as swing factors against microbes. Mol Plant. 2024;17:1341–3 39155502 10.1016/j.molp.2024.08.005

[45] Bulgarelli D, Garrido-Oter R, Münch PC. et al. Structure and function of the bacterial root microbiota in wild and domesticated barley. Cell Host Microbe. 2015;17:392–403 25732064 10.1016/j.chom.2015.01.011 PMC4362959

[46] Ping X, Khan RAA, Chen S. et al. Deciphering the role of rhizosphere microbiota in modulating disease resistance in cabbage varieties. Microbiome. 2024;12:160 39215347 10.1186/s40168-024-01883-0 PMC11363401

[47] Xia X, Wei Q, Wu H. et al. *Bacillus* species are core microbiota of resistant maize cultivars that induce host metabolic defense against corn stalk rot. Microbiome. 2024;12:156 39180084 10.1186/s40168-024-01887-w PMC11342587

[48] Banerjee S, Schlaeppi K, Van Der Heijden MGA. Keystone taxa as drivers of microbiome structure and functioning. Nat Rev Microbiol. 2018;16:567–76 29789680 10.1038/s41579-018-0024-1

[49] Li H, Luo Q-P, Zhao S. et al. Effect of phenol formaldehyde-associated microplastics on soil microbial community, assembly, and functioning. J Hazard Mater. 2023;443:130288 36335899 10.1016/j.jhazmat.2022.130288

[50] Du Y, Han X, Tsuda K. Microbiome-mediated plant disease resistance: recent advances and future directions. J Gen Plant Pathol. 2024;91:1–17

[51] Fira D, Dimkić I, Berić T. et al. Biological control of plant pathogens by *Bacillus* species. J Biotechnol. 2018;285:44–55 30172784 10.1016/j.jbiotec.2018.07.044

[52] Dos Santos IB, Pereira APDA, De Souza AJ. et al. Selection and characterization of *Burkholderia* spp. for their plant-growth promoting effects and influence on maize seed germination. *Frontiers*. Soil Sci. 2022;1:805094

[53] Akaniro IR, Chibuike IV, Onwujekwe EC. et al. *Penicillium* species as chassis for biomanufacturing and environmental sustainability in the modern era: progress, challenges, and future perspective. Fungal Biology Reviews. 2023;46:100326

[54] Sánchez-Cañizares C, Jorrín B, Poole PS. et al. Understanding the holobiont: the interdependence of plants and their microbiome. Curr Opin Microbiol. 2017;38:188–96 28732267 10.1016/j.mib.2017.07.001

[55] Chen Y, Bonkowski M, Shen Y. et al. Root ethylene mediates rhizosphere microbial community reconstruction when chemically detecting cyanide produced by neighboring plants. Microbiome. 2020;8:4 31954405 10.1186/s40168-019-0775-6 PMC6969408

[56] Chen T, Nomura K, Wang X. et al. A plant genetic network for preventing dysbiosis in the phyllosphere. Nature. 2020;580:653–7 32350464 10.1038/s41586-020-2185-0 PMC7197412

[57] Liu C, Geng HY, Li WX. et al. Innate root exudates contributed to contrasting coping strategies in response to *Ralstonia solanacearum* in resistant and susceptible tomato cultivars. J Agric Food Chem. 2023;71:20092–104 38051256 10.1021/acs.jafc.3c06410

[58] Park JH, Choi GJ, Lee HB. et al. Griseofulvin from *Xylaria* sp. strain F0010, an endophytic fungus of *Abies holophylla* and its antifungal activity against plant pathogenic fungi. J Microbiol Biotechnol. 2005;15:112–7

[59] Huang H, Li D, Jiang S. et al. Integrated transcriptome and proteome analysis reveals that the antimicrobial griseofulvin targets *Didymella segeticola* beta-tubulin to control tea leaf spot. Phytopathology. 2023;113:194–205 36173282 10.1094/PHYTO-02-22-0061-R

[60] Massadeh AM, Alhusban AA. A developing method for preconcentration and determination of Pb, Cd, Al and As in different herbal pharmaceutical dosage forms using chelex-100. Chem Pap. 2021;75:3563–73

[61] Tamura K, Stecher G, Kumar S. MEGA11: molecular evolutionary genetics analysis version 11 (FU Battistuzzi, Ed.). Mol Biol Evol. 2021;38:3022–7 33892491 10.1093/molbev/msab120 PMC8233496

[62] Edgar RC . UPARSE: highly accurate OTU sequences from microbial amplicon reads. Nat Methods. 2013;10:996–8 23955772 10.1038/nmeth.2604

[63] Bokulich NA, Kaehler BD, Rideout JR. et al. Optimizing taxonomic classification of marker-gene amplicon sequences with QIIME 2’s q2-feature-classifier plugin. Microbiome. 2018;6:90 29773078 10.1186/s40168-018-0470-z PMC5956843

[64] Zhang Z, Yang S, Zhan Y. et al. Tomato microbiome under long-term organic and conventional farming. iMeta. 2022;1:e48 38868718 10.1002/imt2.48 PMC10989780

[65] Liu X, Yang C, Dong H. et al. TaRLK2.4, a transgressive expression receptor like kinase, improves powdery mildew resistance in wheat. Int J Biol Macromol. 2024;277:134387 39111505 10.1016/j.ijbiomac.2024.134387

