# Supplementary material for: Metabolite-mediated responses of phyllosphere microbiota to powdery mildew infection in resistant and susceptible black currant cultivars
Source: Hortic Res. 2025 Mar 25;12(7):uhaf092. doi: 10.1093/hr/uhaf092 (PMC12077297; doi:10.1093/hr/uhaf092)
Supplement: Web_Material_uhaf092 [file web_material_uhaf092.zip › New-Supplementary material-Figures.docx]

**Supplementary material**


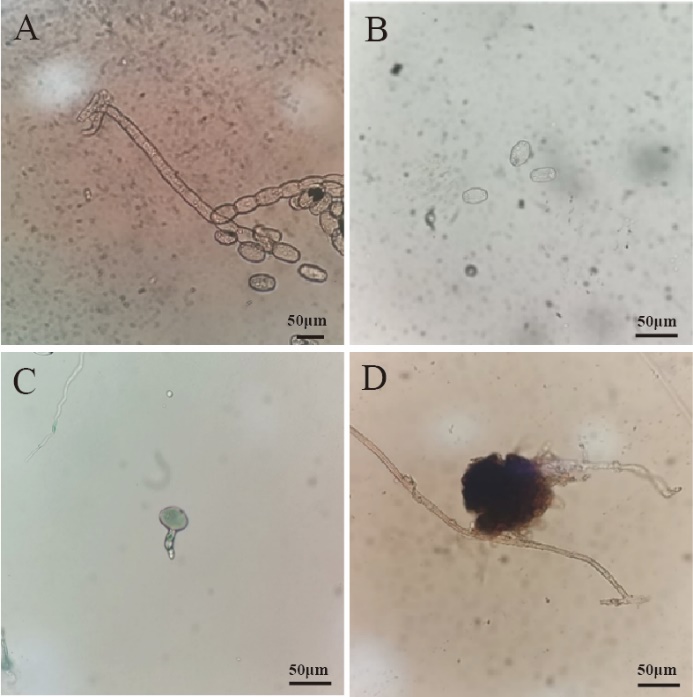


**Supplementary Figure S1.** Morphological characteristics of fungus causing powdery mildews on Black currant. (A) Conidiophore, (B) Conidia, (C) Germinating conidia, (D) Cleistothecium.


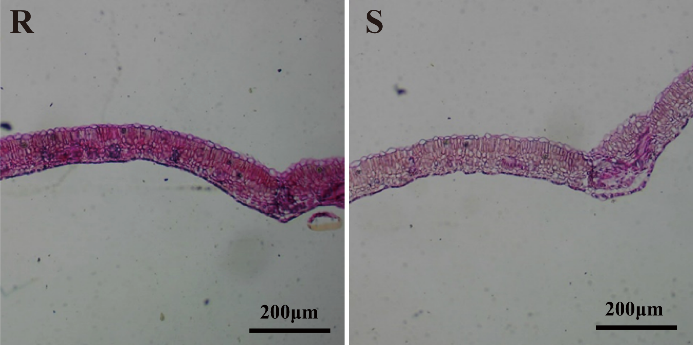


**Supplementary Figure S2.** Leaf cross section (10x). R: resistant cultivar ‘16A’, S: susceptible cultivar ‘Bright leaf’.


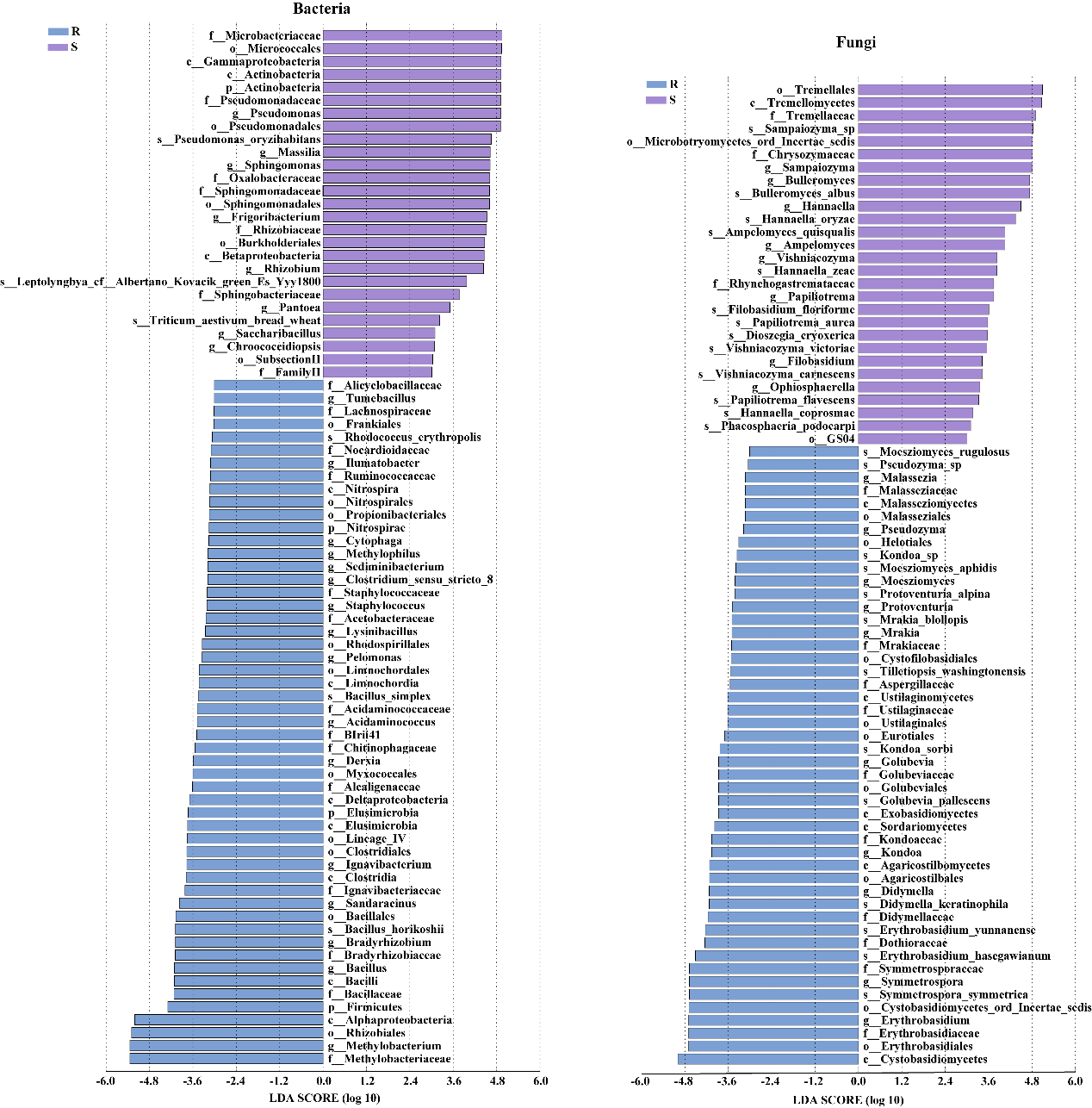


**Supplementary Figure S3.** Linear discriminant analysis (LDA) scores from the LDA effect size (LEfSe). The taxa displayed in the figure are those with an absolute LDA score > 3.


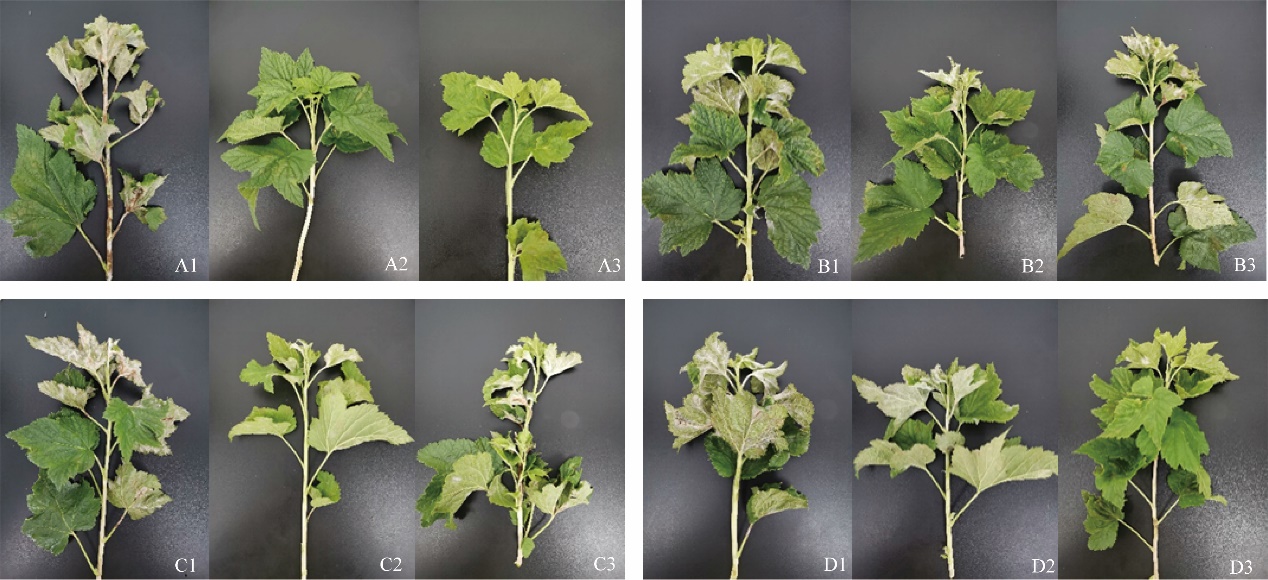


**Supplementary Figure S4.** Phenotypic comparison of spraying three metabolites. (A1) Spraying water on susceptible cultivar. (A2) Spraying water on resistant cultivar. (A3) Spraying triadimefon on susceptible cultivar. (B1-B3) Spraying 50mg/L, 100mg/L, 150mg/L of salicylic acid on susceptible cultivar. (C1-C3) Spraying 5mg/L, 10mg/L, 15mg/L of trans-zeatin on susceptible cultivar. (D1-D3) Spraying 50mg/L, 100mg/L, 150mg/L of griseofulvin on susceptible cultivar.


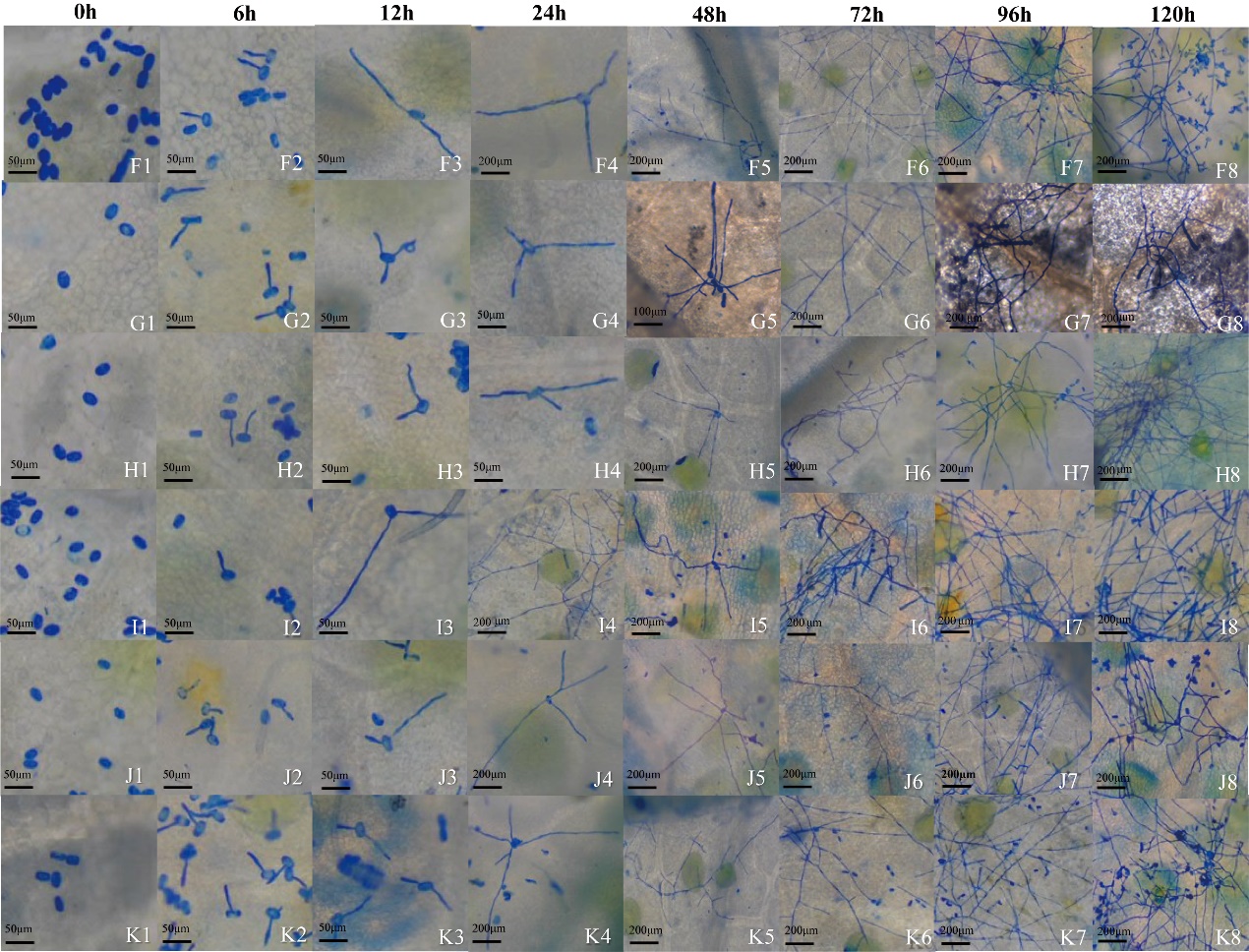


**Supplementary Figure S5.** Spore growth characters with spraying water and different concentrations salicylic acid and trans-zeatin, at 0, 6, 12, 24, 48, 72, 96 and 120 h after PM pathogen inoculation. (F1-F8) Spraying 5 mg/L trans-zeatin on susceptible cultivar. (G1-G8) Spraying 10 mg/L trans-zeatin on susceptible cultivar. (H1-H8) Spraying 15 mg/L trans-zeatin on susceptible cultivar. (I1-I8) Spraying 50 mg/L salicylic acid on susceptible cultivar. (J1-J8) Spraying 100 mg/L salicylic acid on susceptible cultivar. (K1-K8) Spraying 150 mg/L salicylic acid on susceptible cultivar.
